# Clinical characteristics and overall survival prognostic nomogram for metaplastic breast cancer

**DOI:** 10.3389/fonc.2023.1030124

**Published:** 2023-03-02

**Authors:** Caihong Zheng, Chengbin Fu, Yahui Wen, Jiameng Liu, Shunguo Lin, Hui Han, Zhonghua Han, Chunsen Xu

**Affiliations:** ^1^ The Graduate School of Fujian Medical University, Fuzhou, Fujian, China; ^2^ Department of Breast Surgery, Fujian Medical University Union Hospital, Fuzhou, Fujian, China; ^3^ Department of General Surgery, Fujian Medical University Union Hospital, Fuzhou, Fujian, China; ^4^ Breast Cancer Institute, Fujian Medical University, Fuzhou, Fujian, China; ^5^ Department of Breast Surgery, Women and Children’s Hospital, School of Medicine, Xiamen University, Xiamen, Fujian, China

**Keywords:** SEER, metaplastic breast cancer, nomogram, overall survival (OS), prognosis

## Abstract

**Background:**

Metaplastic breast cancer (MBC) is a rare breast tumor and the prognostic factors for survival in patients still remain controversial. This study aims to develop and validate a nomogram to predict the overall survival (OS) of patients with MBC.

**Methods:**

We searched the Surveillance, Epidemiology, and End Results (SEER) database for data about patients including metaplastic breast cancer and infiltrating ductal carcinoma (IDC) from 2010 to 2018. The survival outcomes of patients between MBC and IDC were analyzed and compared with the Kaplan-Meier (KM) method. MBC patients were randomly allocated to the training set and validation I set by a ratio of eight to two. Meanwhile, the performance of this model was validated again by the validation II set, which consisted of MBC patients from the Union Hospital of Fujian Medical University between 2010 and 2018. The independent prognostic factors were selected by univariate and multivariate Cox regression analyses. The nomogram was constructed to predict individual survival outcomes for MBC patients. The discriminative power, calibration, and clinical effectiveness of the nomogram were evaluated by the concordance index (C-index), the receiver operating characteristic (ROC) curve, and the decision curve analysis (DCA).

**Results:**

MBC had a significantly higher T stage (T2 and above accounting for 75.1% vs 39.9%), fewer infiltrated lymph nodes (N0 accounted for 76.2% vs 67.7%), a lower proportion of ER (22.2% vs 81.2%), PR (13.6% vs 71.4%), and HER-2(6.7% vs 17.7%) positive, radiotherapy(51.6% vs 58.0%) but more chemotherapy(67.5% vs 44.7%), and a higher rate of mastectomy(53.2% vs 36.8%), which was discovered when comparing the clinical baseline data between MBC and IDC. Age at diagnosis, T, N, and M stage, as well as surgery and radiation treatment, were all significant independent prognostic factors for overall survival (OS). In the validation I cohort, the nomogram’s C-index (0.769 95% CI 0.710 -0.828) was indicated to be considerably higher than the standard AJCC model’s (0.700 95% CI 0.644 -0.756). Nomogram’s great predictive capability capacity further was supported by the comparatively high C-index of the validation II sets (0.728 95%CI 0.588-0.869).

**Conclusions:**

Metaplastic breast cancer is more aggressive, with a worse clinical prognosis than IDC. This nomogram is recommended for patients with MBC, both American and Chinese, which can help clinicians make more accurate individualized survival analyses.

## Background

Female breast cancer has overtaken others as the most commonly diagnosed malignancy, with an expected 2.3 million new cases in 2020, based on data from the International Agency for Research on Cancer (1). Metaplastic breast cancer (MBC) is a group of rare and heterogeneous invasive carcinomas, characterized by cell differentiation of the tumor epithelium towards squamous and/or mesenchymal-like components such as spindle cells, chondrocytes, and osteoblasts, accounting for only 0.2-5% of all breast cancer (2). MBC has been considered more aggressive, with poor clinical outcomes and a large unmet demand for treatment, compared to invasive ductal breast carcinoma(IDC). Due to the rarity of MBC, limitations of tailored understanding of the clinical characteristics and prognosis exist in previous reports. The majority of MBC’s local and system-optimally regulated treatment approaches are deduced from IDC’s treatment practice and have not been rigorously confirmed in MBC patients. The American Joint Committee on Cancer Staging (AJCC) system is the most commonly used to assess a patient’s prognosis for breast cancer (3). However, disregard for other parameters (such as age), limited precision, and poor performance in forecasting individual survival risk are some of its main disadvantages. Patients with MBC, therefore, require a tailor-made prediction model. Nomogram is confirmed as a reliable and alternative prognosis assessment tool in many carcinomas and is even thought to be a new emerging standard (4). Based on clinical, immunological, and pathological data from the Surveillance, Epidemiology, and End Results (SEER) database, we intend to develop a maneuverable, definitive, and high-exactness nomogram to foresee MBC patient individual survival endings (5–7).

## Materials and methods

### Data source and study population

The Surveillance, Epidemiology, and End Results (SEER) Program provides information on cancer statistics in an effort to reduce the cancer burden, and no ethics committee review approval was needed. We included patients diagnosed with confirmed MBC by extracting and screening data from the SEER database, which included persons from 18 areas (1975-2018) and was released on August 20, 2021. And patients diagnosed with confirmed MBC from the Union Hospital of Fujian Medical University between 2010 and 2018, also were included in this study. The including and excluding criteria of patients with MBC were as follows.

Inclusion criteria:

(1) the years of diagnosis spanned from 2010 to 2018.
(2) the primary site of the tumor was the breast.
(3) according to ICD-0-3, histological types were restricted to 8500/3 (IDC) and 8052/3, 8070/3-8072/3, 8074/3, 8560/3, 8571/3,8572/3, 8575/3, 8980/3 (MBC) (8).

Exclusion criteria:

(1) patients with missing information of age at diagnosis, marital status, PR status, ER status, HER2 status, surgery, or other important clinicopathological data.
(2) patients under the age of 18 years old.
(3) the patients have other cancer other than breast cancer.
(4) patients who have survived or followed up less than one month since the initial diagnosis.
(5) diagnosis of MBC patients obtained from autopsy or death.

The demographic parameters included age at diagnosis is distributed into <50 years, 50-64 years, 65-79 years, and 80+ years, gender is divided into women and males, race (white, black, and others), marital status is classified into married, single and divorced (separated, widowed and divorced). The clinicopathologic parameters included laterality of primary is divided into right and left, site of the tumor is distributed into 502, 503, 504,505 and others, AJCC stage is divided into I, II, III, and IV, T stage is divided intoT1, T2, T3, and T4, N stage is divided into N0, N1, N2, and N3), M stage is divided intoM0 and M1, ER status is distributed into negative or positive), PR status is distributed into negative or positive, HER2 status is distributed into negative or positive, the subtype of breast cancer is distributed into HR+/HER2-, HR+/HER2+, HR-/HER2+, and HR-/HER2-, surgery type is classified into no surgery, breast-conserving, and mastectomy, radiotherapy is divided into yes and no, and chemotherapy is divided into yes and no. The primary clinical outcome for this series was overall survival (OS), which was defined as the time from the date of diagnosis to the date of death owing to any cause or the final follow-up.

### Statistical analysis

The chi-square test or Fisher exact test was performed to evaluate the clinical and pathological characteristics of the different cohorts. Kaplan-Meier analysis was utilized to construct the survival curve. The discrepancy in the survival of each group was evaluated using the log-rank test. The life table approach was performed to figure out overall survival over three and five years. The patients with MBC were split into the training sets and validation sets with an 8:2 ratio, using the “createDataPartition” function of R software to guarantee that result events were distributed randomly. The Cox regression model, hazard ratios (HRs), and 95% confidence intervals (CIs) were utilized to confirm prognostic factors in the training set. Univariate Cox regression analyses were conducted for all variables, followed by multivariate Cox regression for variables with *p* < 0.1 in univariate Cox regression. Finally, variables with *p* < 0.05 in multivariate Cox backward stepwise regression were determined as independent risk factors. To prevent multicollinearity, in the multivariate analysis, T, N, and M stage variables were utilized instead of AJCC stage variables. Based on the findings of the multivariate Cox regression, the nomogram model was generated utilizing the RMS package in the R program, and further verified by the validation sets. The C-index, the receiver operating characteristic (ROC) curve, and the decision curve analysis (DCA) were used to evaluate the predictive accuracy, discrimination ability, as well as clinical effectiveness and benefit of the nomogram model respectively (9, 10).

The SEERStat software, version 8.3.9, was applied to extract the data. R software version 3.5.3 and IBM SPSS Statistics 26 were utilized to conduct statistical analyses. For all of the analyses, a two-tailed *p*-value of <0.05 was deemed statistically significant.

## Results

### Clinical and pathological characteristics

A total of 225,548 eligible patients were included in this study, based on data from the SEER database. The median age of 223943(99.3%) IDC patients was 59 years old, whereas 1605 MBC patients had a median age of 61 years old. The proportion of MBC patients over 65 years old was greater than the proportion of IDC patients (*p <*0.001). The proportion of black patients with MBC is larger (*p <*0.001). When it comes to marital status, MBC patients have a higher number of divorced patients than IDC patients, but a lower proportion of married patients. Compared to patients with IDC, those with MBC had considerably significantly larger primary tumors. Furthermore, MBC patients exhibited a greater T stage than IDC patients (*p <*0.001), with T2 (47.9% vs 30.9%), T3 (16.9% vs 5.1%), and T4 (10.3% vs 3.9%), but a lower axillary lymph node involvement rate (76.2% vs. 67.7%, *p <*0.001), as well as no significant difference in the proportion of distant metastasis(4.9% vs 4.0%, *p*=0.066). meanwhile, the majority of MBC patients are “triple-negative”, with HR-/HER2- (68.3% vs 12.4% *p*<0.001), a meaning lower expression of the ER (22.2% vs 81.2%), PR (13.6% vs 71.4%), and HER-2(6.7% vs 17.7%) receptors (*p*<0.001). MBC patients received less radiotherapy but more chemotherapy. Patients with MBC were more likely to have a mastectomy (53.2% vs 36.8%), whereas those with IDC were more likely to have breast-conserving (41.9% vs 57.4%)surgery(*p*<0.001) (**Table 1**). There were no statistically significant differences between the training and verification I sets of MBC patients with 17 variables (**Supplementary Table 1**).

**Table 1:** The characteristics of 225,548 breast cancer patients.

Characteristic	MBC, N (%)	IDC, N (%)	N (%)	*P*-value
	1605(0.7%)	223943(99.3%)	225548(100%)	
Age(years)				< 0.001
<50	349(21.7%)	54559(24.4%)	54908(24.3%)	
50-64	586(36.5%)	88196(39.3%)	88782(39.4%)	
65-79	471(29.4%)	64271(28.7%)	64742(28.7%)	
80+	199(12.4%)	16917(7.6%)	17116(7.6%)	
Sex				0.137
Female	1598(99.6%)	222244(99.2%)	223842(99.2%)	
Male	7(0.4%)	1699(0.8%)	1706(0.8%)	
Race				< 0.001
White	1218(75.9%)	175687(78.5%)	176905(78.4%)	
Black	265(16.5%)	24573(10.9%)	24838(11.0%)	
Others	122(7.6%)	23683(10.6%)	23805(10.6%)	
Marital				< 0.001
Married	843(52.5%)	132731(59.3%)	133574(59.2%)	
Single	305(19.0%)	36779(16.4%)	37084(16.5%)	
Divorced	457(28.5%)	54433(24.3%)	54890(24.3%)	
Laterality				0.745
Right	799(49.8%)	110570(49.4%)	111369(49.4%)	
Left	806(50.2%)	113373(50.6%)	114179(50.6%)	
Site				0.461
others	625(38.9%)	85240(38.1%)	85865(38.1%)	
502	210(13.1%)	29356(13.1%)	29566(13.1%)	
503	95(5.9%)	12800(5.7%)	12895(5.7%)	
504	538(33.5%)	79353(35.4%)	79891(35.4%)	
505	137(8.5%)	17194(7.7%)	17331(7.7%)	
AJCC stage				< 0.001
I	359(22.4%)	115640(51.6%)	115999(51.4%)	
II	922(57.4%)	76361(34.1%)	77283(34.3%)	
III	246(15.3%)	23071(10.3%)	23317(10.3%)	
IV	78(4.9%)	8871(4.0%)	8949(4.0%)	
T stage				< 0.001
T1	399(24.9%)	134484(60.1%)	134883(59.8%)	
T2	770(47.9%)	69194(30.9%)	69964(31.0%)	
T3	271(16.9%)	11600(5.1%)	11871(5.3%)	
T4	165(10.3%)	8665(3.9%)	8830(3.9%)	
N stage				< 0.001
N0	1223(76.2%)	151690(67.7%)	152913(67.8%)	
N1	276(17.2%)	54122(24.2%)	54398(24.1%)	
N2	71(4.4%)	11592(5.2%)	11663(5.2%)	
N3	35(2.2%)	6539(2.9%)	6574(2.9%)	
M stage				0.066
M0	1527(95.1%)	215072(96.0%)	216599(96.0%)	
M1	78(4.9%)	8871(4.0%)	8949(4.0%)	
ER status				< 0.001
Negative	1248(77.8%)	42078(18.8%)	43326(19.2%)	
Positive	357(22.2%)	181865(81.2%)	182222(80.8%)	
PR status				< 0.001
Negative	1387(86.4%)	64097(28.6%)	65484(29.0%)	
Positive	218(13.6%)	159846(71.4%)	160064(71.0%)	
HER-2 status				< 0.001
Negative	1497(93.3%)	184336(82.3%)	185833(82.4%)	
Positive	108(6.7%)	39607(17.7%)	39715(17.6%)	
Subtype				< 0.001
HR+/HER2-	401(25.0%)	156663(70.0%)	157064(69.6%)	
HR+/HER2+	37(2.3%)	27758(12.4%)	27795(12.3%)	
HR-/HER2+	71(4.4%)	11849(5.3%)	11920(5.3%)	
HR-/HER2-	1096(68.3%)	27673(12.4%)	28769(12.8%)	
Surgery				< 0.001
no surgery	78(4.9%)	12886(5.8%)	12964(5.8%)	
breast-conserving	673(41.9%)	128573(57.4%)	129246(57.3%)	
mastectomy	854(53.2%)	82484(36.8%)	83338(36.9%)	
Chemotherapy				< 0.001
No	521(32.5%)	123815(55.3%)	124336(55.1%)	
Yes	1084(67.5%)	100128(44.7%)	101212(44.9%)	
Radiotherapy				< 0.001
No	777(48.4%)	93995(42.0%)	94772(42.0%)	
Yes	828(51.6%)	129948(58.0%)	130776(58.0%)	

MBC Metaplastic breast carcinoma, IDC Infiltrating ductal carcinoma, 502 Upper-inner quadrant of breast, 503 Lower-inner quadrant of breast, 504 Upper-outer quadrant of breast, 505 Lower-outer quadrant of breast, ER, Estrogen receptor; PR, Progesterone receptor; HER-2, Human epidermal growth factor receptor 2.

For the validation II cohort, 49 Chinese MBC patients, who were diagnosed in the Union Hospital of Fujian Medical University between 2010 and 2018, were included in this study. Among these patients, the median age was 50 years old, and the median follow-up time was 79 months (3-139 months). When it comes to marital status, Chinese MBC patients have a higher number of married patients. Compared to American MBC patients, a higher proportion of Chinese MBC patients were under 65 years old, with particularly less than 50 years old(20.8% vs 51.0%). Furthermore, a higher percentage of mastectomy (95.9%) was performed on Chinese MBC patients. And in comparison with American patients, a larger proportion of Chinese patients with MBC receive chemotherapy (67.0% vs 95.9%) and less radiotherapy (50.6% vs 38.8%). There were no statistically significant differences between American patients and Chinese patients with MBC on other variables (**Supplementary Table 2**).

### Survival analysis

**Figure 1:**
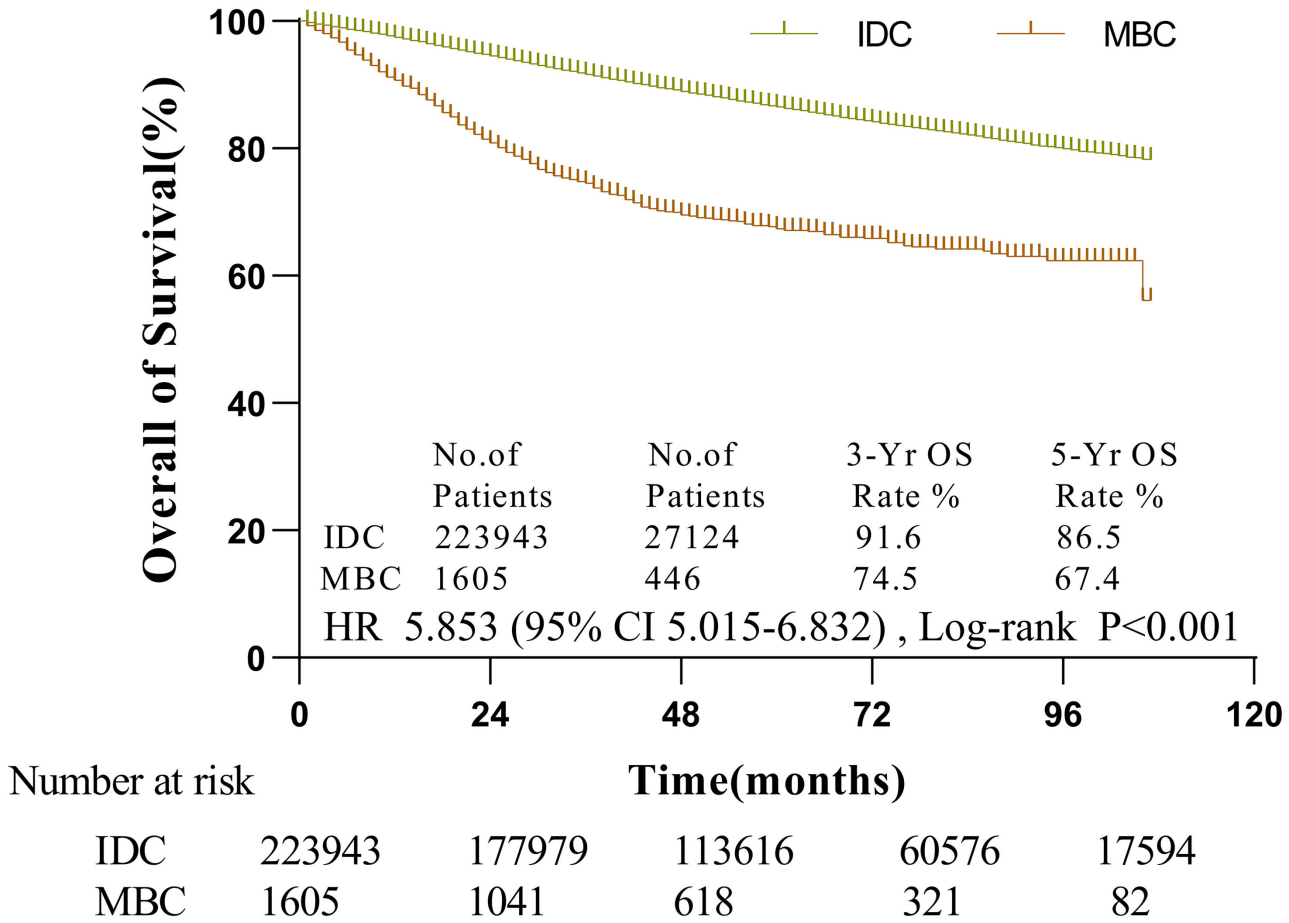
The survival of patients with MBC and IDC by Kaplan-Meier analysis. Patients with MBC had worse survival (HR = 5.853, 95% CI, 5.015-6.832,p < 0.001) with 3- and 5-year OS rates of 74.5 and 67.4% vs. 91.6 and 86.5% in IDC patients, respectively.

The median follow-up period of MBC was 53 months (1-107 months). According to the KM analysis, MBC patients’ survival was considerably shortened than that of IDC patients (*p* < 0.001). The three-year and five-year overall survival rates of MBC were 74.5 and 67.4%, respectively. Likewise, IDC’s three-year and five-year overall survival rates were 91.6 and 86.5%, respectively (**Figure 1**). The histological category of MBC was found to be a poorer prognosis element for breast carcinoma by univariate Cox regression analysis

### Prognostic factors in MBC

The patients with MBC were split into the training sets (n =1284) and validation I sets (n = 321) with an 8:2 ratio, using the “createDataPartition” function of R software to guarantee that result events were distributed randomly.

**Figure 2:**
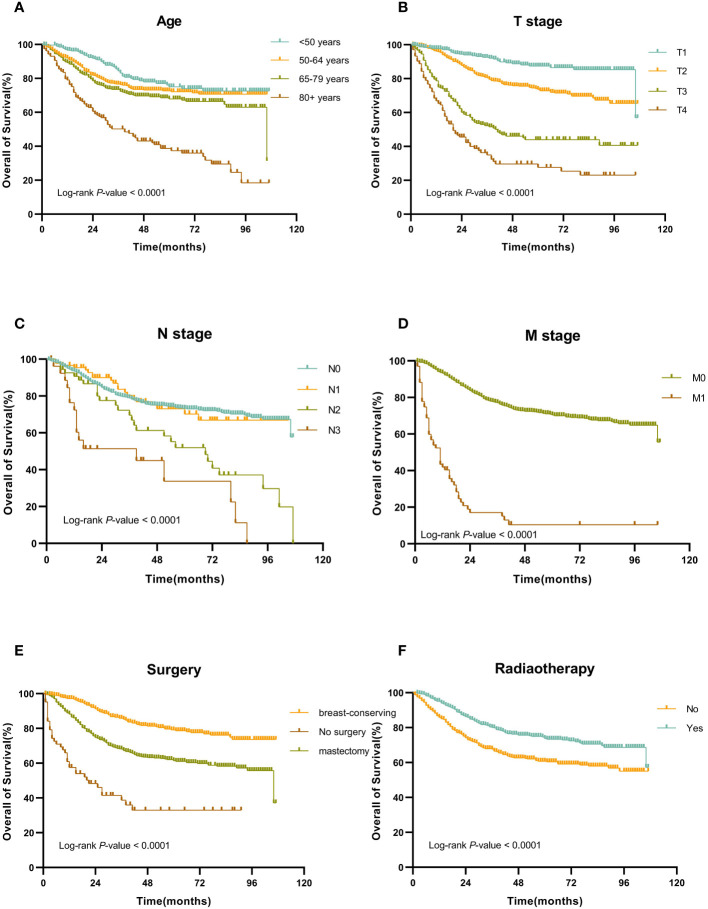
Kaplan-Meier OS curves for patients with MBC according to different independent prognostic factors. **(A-F)** Kaplan-Meier OS curves for patients with MBC according to **(A)** age, **(B)** T stage, **(C)** N stage, **(D)** M stage, **(E)** surgery, and **(F)** surgery.

In the training set, the Cox regression model was utilized to find the variables that influence MBC prognosis. Age, marital status, tumor site, AJCC stage, T stage, N stage, M stage, surgery, radiotherapy, and chemotherapy all demonstrated statistically significant variations in survival prognostic variables, in the univariate analysis, but the sex (*p*=0.700), race (*p*=0.131), PR status (*p*=0.312), ER status (*p*=0.296), HER-2 status(p=0.518) and subtype (*p*=0.913). Finally, followed by multivariate Cox regression, age, T stage, N stage, M stage, surgery, and radiotherapy were determined as independent prognostic factors for patients with MBC (**Table 2**). Kaplan-Meier survival curves of each independent prognostic factor were shown in **Figure 2**.

**Table 2:** Univariate and multivariate Cox regression analysis of overall survival (MBC Training Cohort).

Characteristics	Univariate analysis		Multivariate analysis	
	HR (95%CI)	*P*-value	HR (95%CI)	*P*-value
Age (years)		**<0.001**		<0.001
<50	Reference		Reference	
50-64	1.37 (1.00-1.91)	0.069	1.44 (1.02-2.02)	0.036
65-79	1.70 (1.21-2.38)	0.002	1.94 (1.38-2.74)	<0.001
80+	4.06 (2.87-5.74)	<0.001	4.26 (2.96-6.15)	<0.001
Sex		0.700		
Female	Reference			
Male	0.68 (0.10-4.85)	0.700		
Race		0.131		
White	Reference			
Black	1.23 (0.95-1.60)	0.120		
Others	0.78 (0.50-1.22)	0.279		
Marital		**<0.001**		
Married	Reference			
Single	1.57 (1.19-2.06)	0.001		
Divorced	1.71 (1.35-2.16)	<0.001		
Laterality		**0.054**		
Right	Reference			
Left	1.23 (1.00-1.51)	0.054		
Site		**0.002**		
others	Reference			
502	0.59 (0.41-0.86)	0.006		
503	0.51 (0.30-0.87)	0.013		
504	0.69 (0.54-0.88)	0.002		
505	0.87 (0.61-1.27)	0.480		
AJCC stage		**<0.001**		
I	Reference			
II	3.29 (2.13-5.07)	<0.001		
III	8.94 (5.67-14.10)	<0.001		
IV	29.35 (18.00-47.92)	<0.001		
T stage		**<0.001**		<0.001
T1	Reference		Reference	
T2	2.28 (1.55-3.35)	<0.001	1.98 (1.34-2.92)	0.001
T3	6.54 (4.40-9.72)	<0.001	4.97 (3.29-7.52)	<0.001
T4	11.16 (7.41-16.81)	<0.001	5.19 (3.29-8.17)	<0.001
N stage		**<0.001**		0.003
N0	Reference		Reference	
N1	2.09 (1.64-2.67)	<0.001	1.37 (1.05-1.79)	0.019
N2	2.73 (1.83-4.07)	<0.001	1.41 (0.92-2.16)	0.110
N3	3.58 (2.19-5.87)	<0.001	2.42 (1.42-4.09)	0.001
M stage		**<0.001**		<0.001
M0	Reference		Reference	
M1	8.46 (6.32-11.32)	<0.001	3.12 (2.21-4.40)	<0.001
ER status		0.296		
Negative	Reference			
Positive	1.14 (0.89-1.45)	0.296		
PR status		0.312		
Negative	Reference			
Positive	0.85 (0.62-1.16)	0.312		
HER-2 status		0.518		
Negative	Reference			
Positive	0.87 (0.56-1.34)	0.518		
Subtype		0.913		
HR+/HER2-	Reference			
HR+/HER2+	0.89 (0.45-1.77)	0.742		
HR-/HER2+	0.81 (0.45-1.48)	0.499		
HR-/HER2-	0.97 (0.76-1.23)	0.792		
Surgery		**<0.001**		<0.001
no surgery	Reference		Reference	
breast-conserving	0.16 (0.11-0.23)	<0.001	0.35 (0.23-0.54)	<0.001
mastectomy	0.34 (0.25-0.51)	<0.001	0.50 (0.35-0.72)	<0.001
Chemotherapy		**<0.001**		
No	Reference			
Yes	0.58 (0.47-0.71)	<0.001		
Radiotherapy		**<0.001**		0.002
No	Reference		Reference	
Yes	0.57 (0.46-0.71)	<0.001	0.69 (0.54-0.87)	0.002

MBC Metaplastic breast carcinoma, 502 Upper-inner quadrant of breast, 503 Lower-inner quadrant of breast, 504 Upper-outer quadrant of breast, 505 Lower-outer quadrant of breast, ER, Estrogen receptor; PR, Progesterone receptor; HER-2, Human epidermal growth factor receptor 2. For all of the analyses, variables with p<0.1 were deemed statistically significant in univariate Cox regression, and variables with p < 0.05 were deemed statistically significant in multivariate Cox.

### Construction and validation of a nomogram

**Figure 3:**
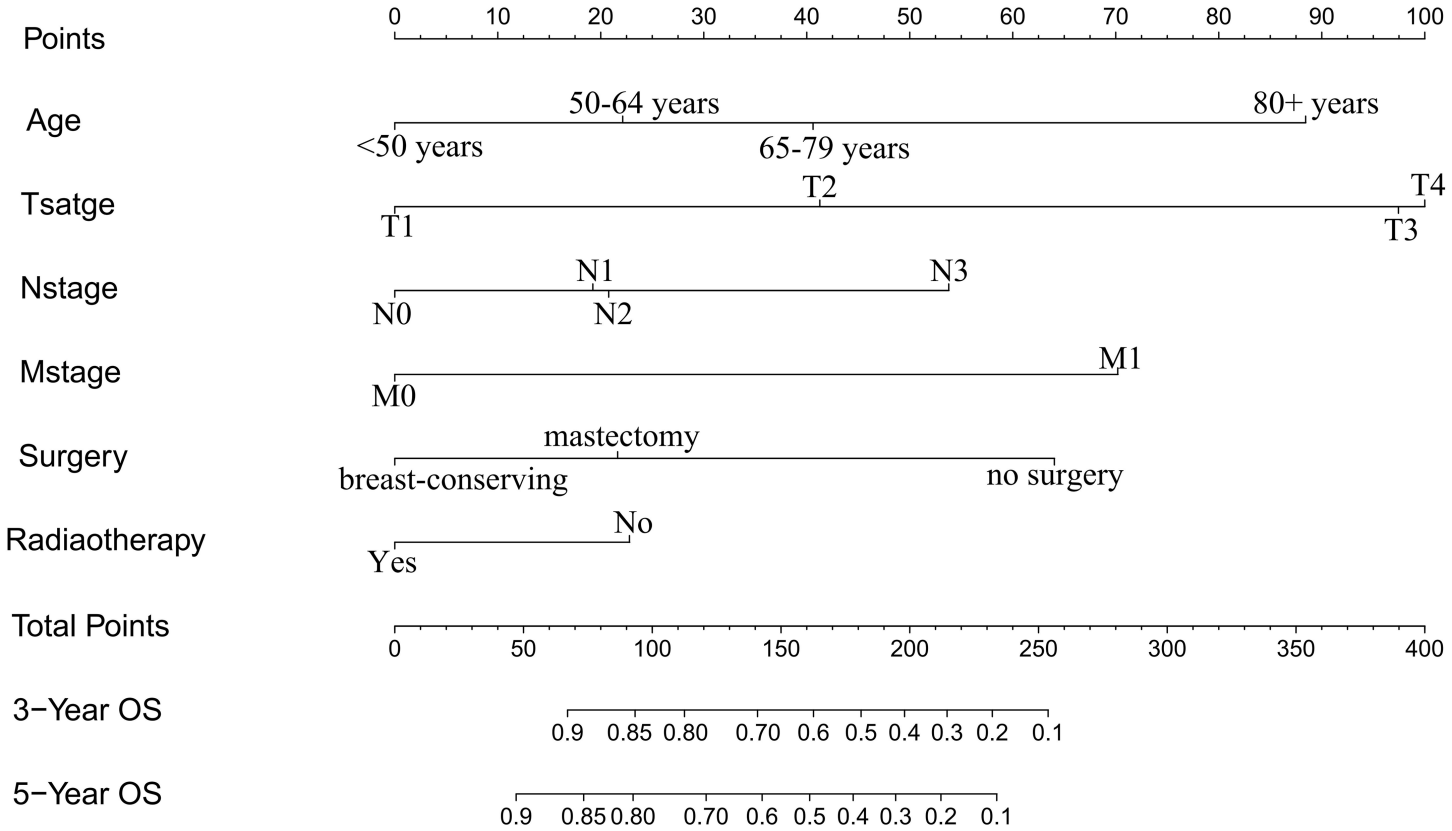
Nomogram predicted 3- and 5-year overall survival for patients with MBC. The nomogram is used by summing the points identified on the top scale for each independent covariate. The total points projected to the bottom scale indicated the % probability of the 3- and 5-year OS.

The independent prognostic factors (age, T stage, N stage, M stage, surgery, and radiotherapy), which were found by the Cox regression, were utilized to develop a nomogram model to assess the overall survival of MBC (**Figure 3**). The nomogram model showed that T stage had the greatest impact on prognosis, and the smallest is radiotherapy. Scores are awarded to all subtypes of all factors (**Table 3**).

**Table 3:** Point assignment and prognostic score in the nomogram (MBC Training Cohort).

Variable	Score
Age(years)	
<50	0
50-64	22
65-79	41
80+	88
T stage	
T1	0
T2	41
T3	97
T4	100
N stage	
N0	0
N1	19
N2	21
N3	54
M stage	
M0	0
M1	70
Surgery	
no surgery	64
breast-conserving	0
mastectomy	22
Radiotherapy	
No	23
Yes	0

**Figure 4:**
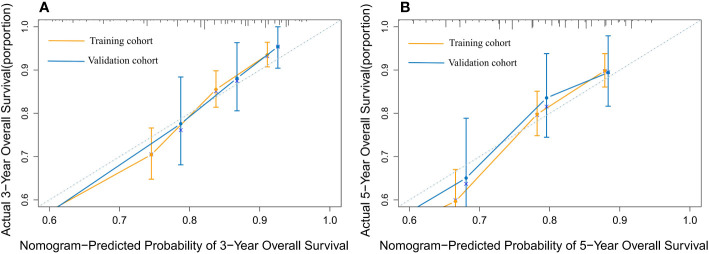
The calibration plot for predicting 3- and 5-year overall survival for patients with MBC. Calibration plot of nomogram prediction of **(A)** 3-year and **(B)** 5-year OS of patients with MBC in the training and validation I sets.

**Figure 5:**
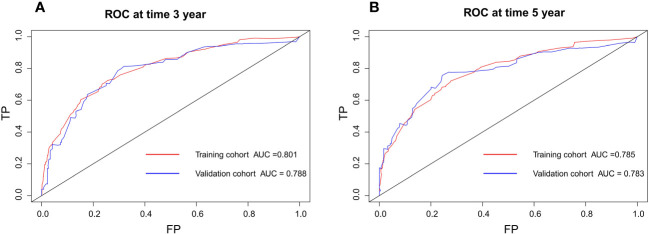
Discriminatory accuracy for predicting OS examined by ROC analysis calculating AUC. There-year OS in the training and validation I sets **(A)**. Five-year OS in the training and validation I sets **(B)**.

**Figure 6:**
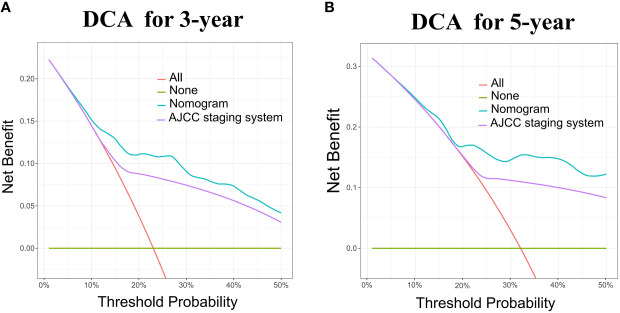
DCA for the Nomogram and AJCC staging system in the validation cohort. DCA in the prediction of patients at 3-year **(A)** and 5-year **(B)** in the training and validation I sets.

The nomogram model has been verified internally and externally. The internal verification revealed that the C-index estimated by overall survival for the training sets was 0.794 (95% CI 0.771-0.816). The C-index indicated by the overall survival of the externally confirmed was 0.769 (95% CI 0.710-0.828), according to the validation I sets. In the training and validation I sets, the calibration plots revealed high uniformity between the nomogram prognostication and the actual observation (**Figure 4**). The ROC of the training and verification I sets is depicted in (**Figure 5**). In the verification I sets, the C-index of the overall survival predicted by the nomogram was 0.769 (95% CI 0.710 -0.828), which was greater than the C-index of the AJCC staging system (C-index=0.700 95% CI 0.644 -0.756). The DCA was applied to make comparisons of the availability and advantages between the nomogram model and the AJCC staging system. In the validation I sets, the nomogram has a greater overall advantage over a number of death hazards than the AJCC staging system, which was revealed by the 3-year and 5-year DCA curves, (**Figure 6**).

**Figure 7:**
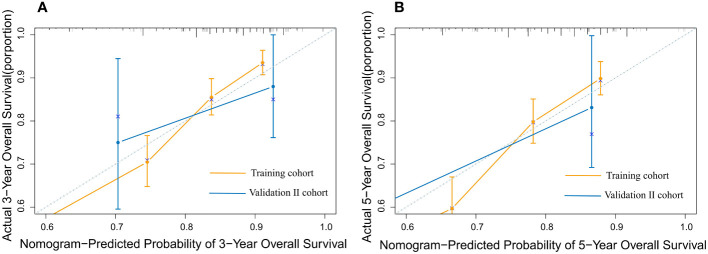
The calibration plot for predicting 3- and 5-year overall survival for patients with MBC. Calibration plot of nomogram prediction of **(A)** 3-year and **(B)** 5-year OS of patients with MBC in the training and validation II sets.

**Figure 8:**
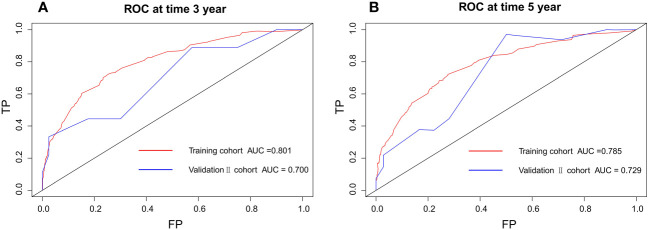
Discriminatory accuracy for predicting OS examined by ROC analysis calculating AUC. There-year OS in the training and validation II sets **(A)**. Five-year OS in the training and validation II sets **(B)**.

**Figure 9:**
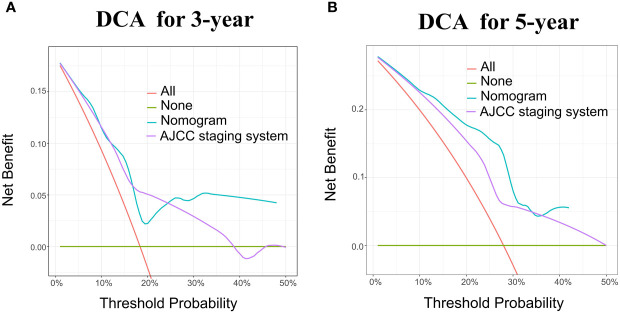
DCA for the Nomogram and AJCC staging system in the validation cohort. DCA in the prediction of patients at 3-year **(A)** and 5-year **(B)** in the training and validation II sets.

According to the validation II sets, the C-index indicated by the overall survival of the externally confirmed was 0.728 (95% CI 0.588-0.869). The calibration plots in the training and validation II sets indicated a comparatively high uniformity between the nomogram prognostication and the actual observation (**Figure 7**). The training and verification II sets’ ROC is provided in **Figure 8**. In the validation II sets, the 3-year and 5-year DCA curves also indicated that the nomogram had a bigger overall advantage over the availability than the AJCC staging scheme (**Figure 9**).

## Discussion

Metaplastic breast cancer is rare and generally highly aggressive invasive carcinoma, accounting for 0.2-5% of all breast cancers, characterized by differentiation of the neoplastic epithelium to squamous and/or mesenchymal components (11). The histologic structure of MBC is diversified, consisting of both neoplastic cells and metaplastic cancer tissue, or just metaplastic neoplastic tissue (12), which was further divided into several subgroups: low-grade adenosquamous carcinoma, fibromatosis-like metaplastic carcinoma, squamous cell carcinoma, metaplastic carcinoma with mesenchymal differentiation, mixed metaplastic carcinoma, according to The World Health Organization (WHO) (2, 11, 13).

Traditionally, for assessing prognosis, diagnosing cancer patients, and selecting the most beneficial treatment modalities, the American Joint Committee on Cancer (AJCC) staging guideline has emerged as the gold standard (3). Given that it ignored other biological factors that impact cancer prognosis, at the level of individual treatment, the decisive status of the AJCC staging system has aroused suspicion. Actually, biological markers and other factors may also play a part. In our study, this nomogram demonstrates that, in addition to the T stage, N stage, and M stage, the age of diagnosis, surgery, and whether radiation is administered have a larger influence on prognosis.

According to earlier research, MBC typically affects women over the age of 50 (2, 11). In our study, age is divided into <50 years old, 50-64 years old, 65-80 years old, and 80+ years old. The univariate and multivariate analysis revealed that age is an independent prognostic factor for patients with MBC. The nomogram model showed that age had a pretty great impact on prognosis, in which the 80+ years old subtype of age is assigned a rather high score.

Currently, ER status, PR status, and HER-2 status are three core indicators in medical decision-making, according to ASCO and NCCN recommendations (14–16). However, in our study, ER,PR, and HER-2 status were not the independent prognostic factor for patients with MBC in either multifactorial or univariate Cox analysis. In this study, the positive rate for these three markers is relatively low. Weigelt et al. also demonstrate that more than 90% of MBC patients have a triple-negative phenotype (13), which is consistent with the findings of this study. Even if HR or HER2 status is positive, the efficacy of endocrine treatment and targeted therapy for MBC patients needs to be further investigated.

MBC manifests as a rapidly increasing palpable breast mass, appearing as an ill-defined phyma on imaging without unique radiological signs (12, 17). In this study, patients with MBC had primary tumors that were noticeably larger than those with IDC. Most MBC patients (75.1%) arrived with tumors that were T2 and above, whereas most IDC patients presented with tumors smaller than 20mm (i.e.T1). Further, T3 and greater stage accounted for 27.2% of MBC, and only 9.2% of IDC (p < 0.001). Interestingly, patients with MBC presented a lower rate of lymph node metastasis (2). In this study, only 23.8% of 1605 patients with MBC demonstrate lymph node involvement. Although lymph node involvement was less frequent, more commonly stage II and above (MBC 77.6% vs. IDC 48.4%, p < 0.0001) were seen in MBC patients. MBC patients were also more likely to have stage III (16.9%) or stage IV disease (10.3%), in comparison to IDC patients (10.3% and 4.0%, respectively). The outcomes of the appeal were also corroborated by single-center data from the Union Hospital of Fujian Medical University.

It was not until 2000 that MBC was officially recognized as a distinct pathologic phenotype (12), which results in lacking randomized controlled studies that evaluate treatment modalities and the prognosis in patients with MBC. And that for patients with MBC, endocrine therapy and targeted therapy, which target ER, PR, and HER2 respectively, have limited benefits. Although poor prognosis indicates the limitations of the existing therapeutic alternatives, like IDC, surgery, radiotherapy, and chemotherapy are still the mainstays of treatment for MBC. No association of surgery type with survival was concluded by Haque W and colleagues, examining patients with MBC from Surveillance, Epidemiology, and End Results (SEER) data from 1988 to 2006 (18). Whereas, in this study, surgery was proven to be an independent prognostic factor for MBC in both univariate and multifactorial analyses. Ninety-four point two percent of MBC and IDC patients were treated surgically, but patients with MBC most frequently underwent mastectomy (53.2%), whereas those with IDC most frequently underwent BCS (57.4%). This discrepancy was attributable to a larger primary tumor of the MBC patients, with 16.9% of MBC primary tumors measuring more than 5cm in size as opposed to just 5.1% of IDC primary tumors. Compared to mastectomy, breast-conserving surgery has a better prognosis, according to Kaplan-Meier overall survival curves for patients with MBC, which may be caused by the effects of receiving radiotherapy following breast-conserving surgery (19), with is consistent with the study of Onitilo and colleagues (20). Meanwhile, the effects of a mastectomy on a patient’s physical appearance, quality of life, and psychological health cannot be denied, which may lead to a poorer prognosis (21).

In addition to surgery, radiotherapy (HR=0.57, p<0.001) was found to be also an independent prognostic factor for MBC in both univariate and multivariate Cox regression analysis. In contrast to pT1-2 N0 instances, radiotherapy was associated with OS improvements in pT3-4/N+ patients (8, 18), according to a report from the National Cancer Data Base (NCDB) (22). And Warren H et al. report that patients at “high risk” who have tumors greater than 5cm in size or more than four metastatic axillary lymph nodes are the ones who can benefit from radiotherapy (8, 22, 23). The majority of the MBC patients in this study have primary tumors that were large enough to benefit from radiation. However, there is a dearth of evidence on the effectiveness of chemotherapy in MBC patients. In this multivariate Cox regression analysis, chemotherapy was proven not to be an independent prognostic factor for MBC patients. The application of chemotherapy is an extension of more prevalent histologic subtypes of breast cancer (24). And retrospective studies by D. Rayson have demonstrated that MBC patients benefit less from conventional chemotherapy regimens than do IDC patients (2).

Notably, studies have found evidence that patients with triple negative breast cancer(TNBC) who have BRCA mutations may benefit from poly(adenosine diphosphate-ribose) polymerase inhibitors (PARPi) and platinum salts treatment. For homologous recombination DNA repair, BRCA1/2 encode proteins are indispensable. And breast cancers with BRCA mutations exist a deficiency in homologous recombination repair. Utilizing the principle of synthetic lethality, the poly(adenosine diphosphate-ribose) polymerase inhibitors (PARPi) could target and kill tumor cells with a deficiency in homologous recombination repair. Therefore, Olaparib is the first PARPi to have received approval for the treatment of breast cancer as a result. On the other hand, BRCA mutations make cancer cells more sensitive to the platinum compound, which is also connected to a defective homologous recombination system. Some findings suggest BRCA mutation carriers had longer disease-free intervals and survival following platinum salt therapy (25–31). Thus, BRCA sequencing could be a suitable biomarker for predicting patient response to PARPi and platinum salts in TNBC. And the majority of MBC patients are “triple-negative”(i.e., negative for human epidermal growth factor receptor 2 and estrogen and progesterone receptors), who may benefit from PARPi and platinum salts treatment.

In contrast to IDC, MBC is a relatively chemorefractory malignancy with a significant unmet demand. Some clinical trials for MBC are now conducted to find more effective treatments. For instance, an isolated study from 2018 discovered a durable response to therapy with a P13K inhibitor (buparlisib) for MBC (32). And a 2018 study observing the response of these MBCs to inhibition of mTOR with Afinitor (everolimus) or Toris (temsirolimus) drugs found that patients with triple-negative MBC treated with mTOR inhibitors warranted further exploration (33, 34). Sylvia Adams et al. also found no additional safety issues in MBC patients treated with the combination of ibritumomab and nabumab, and achieved an objective remission rate of 18% for the primary endpoint (35). Meanwhile, in examining the levels of tumor-infiltrating lymphocytes (TILs) and survival data of patients with MBC, Kalaw et al. found the clinical significance and prognostic value of FOXP3, PD-1/PD-L1 and tumor-infiltrating lymphocytes in MBC and confirmed that immunotherapy may be a potential treatment for part patients with MBC (36).

The nomogram model has been validated internally and externally in multiple ways. A relatively higher C-index, relatively high uniformity of the calibration plots, a great receiver operating characteristic(ROC) curve, and decision curve analysis(DCA), prove that the nomogram model has higher predictive accuracy, stronger discriminative ability, greater clinical effectiveness, and benefit respectively. And the nomogram model had a bigger overall advantage over the availability than the AJCC staging model.

In this study, the nomogram model could also be applied to the Chinese MBC patients, which was confirmed by the verification II sets. According to the data of 49 MBC patients from the Union Hospital of Fujian Medical University, Chinese MBC patients more received a mastectomy, with a larger proportion of chemotherapy and less radiotherapy. Part of the reason is that patients with MBC have a younger age composition in China. Young breast cancer patients have distinctive biological behavior, a more aggressive type of pathology, and are more likely to accept a mastectomy and chemotherapy, which was also proved by Partridge et al. (37).

Limitations of this study include that we failed to explore the characteristics and prognosis of several subgroups of MBC separately. Second, SEER data lacks information about BRCA, FOXP3, PD-1/PD-L1, chemotherapy regimens, and genomic profiling. Additionally, there is a dearth of more data from Chinese research centers to verify the nomogram. Finally, prospective research on therapy options and prognosis is critical, although MBC is relatively rare. However, our investigation provided fresh insight into the clinicopathological features and prognosis of MBC patients.

## Conclusions

MBC patients have larger primary tumors, less lymph node invasion, mostly triple-negative phenotype, and relatively chemorefractory tumors with a high unmet need. Patients with MBC have a much poorer prognosis than those with IDC. This nomogram is recommended for patients with MBC, both American and Chinese, which can help clinicians make more accurate individualized survival analyses.

## Data availability statement

The original contributions presented in the study are included in the article/**Supplementary Material**. Further inquiries can be directed to the corresponding authors.

## Ethics statement

The studies involving human participants were reviewed and approved by the Union Hospital of Fujian Medical University. The patients/participants provided their written informed consent to participate in this study.

## Author contributions

Conception and design: CZ and CX; Development of methodology: CZ, CF, YW, JL, SL, HH, ZH, and CX; Acquisition of data, analysis, and interpretation of data (e.g., statistical analysis, biostatistics, computational analysis): CZ, CF, ZH, and CX; Writing, review and/or revision of the manuscript: CZ, CF, ZH, and CX; Study supervision: SL and HH; Revising: CZ, CF, ZH, and CX. All authors contributed to the article and approved the submitted version.
