# Supplementary material for: Clinical characteristics and overall survival prognostic nomogram for metaplastic breast cancer
Source: Front Oncol. 2023 Mar 2;13:1030124. doi: 10.3389/fonc.2023.1030124 (PMC10018193; doi:10.3389/fonc.2023.1030124)
Supplement: Supplementary file 1 [file Table_1.doc]

| **Supplementary Table 1.** The characteristics of 1605 patients of MBC | | | | | | | |
| --- | --- | --- | --- | --- | --- | --- | --- |
| **Characteristic** | **Training cohort,N(%)** |  | **Validation cohort,N(%)** |  | **N(%)** |  | ***P***-value |
|  | 1284(80.0%) |  | 321(20.0%) |  | 1605(100.0%) |  |  |
| Age(years) |  |  |  |  |  |  | 0.273 |
| <50 | 267（20.8%） |  | 82 (25.5%) |  | 349(21.7%) |  |  |
| 50-64 | 474（36.9%） |  | 112(34.9%) |  | 586(36.5%) |  |  |
| 65-79 | 385（30.0%） |  | 86(26.8%) |  | 471(29.3%) |  |  |
| 80+ | 158（12.3%） |  | 41(12.8%) |  | 199(12.5%) |  |  |
| Sex |  |  |  |  |  |  | 0.705 |
| Female | 1278（99.5%） |  | 320(99.7%) |  | 1598(99.6%) |  |  |
| Male | 6（0.5%） |  | 1 (0.3%) |  | 7(0.4%) |  |  |
| Race |  |  |  |  |  |  | 0.602 |
| White | 968（75.4%） |  | 250(77.9%) |  | 1218(75.9%) |  |  |
| Black | 215（16.7%） |  | 50(15.6%) |  | 265(16.5%) |  |  |
| Others | 101（7.9%） |  | 21(6.5%) |  | 122(7.6%) |  |  |
| Marital |  |  |  |  |  |  | 0.805 |
| Married | 673（52.4%） |  | 170(53.0%) |  | 843(52.5%) |  |  |
| Single | 248（19.3%） |  | 57(17.8%) |  | 305(19.0%) |  |  |
| Divorced | 363（28.3%） |  | 94(29.2%) |  | 457(28.5%) |  |  |
| Laterality |  |  |  |  |  |  | 0.091 |
| Right | 626（48.8%） |  | 173(53.9%) |  | 799(49.8%) |  |  |
| Left | 658（51.2%） |  | 148(46.1%) |  | 806(50.2%) |  |  |
| Site |  |  |  |  |  |  | 0.461 |
| others | 502（39.1%） |  | 123(38.3%) |  | 265(38.9%) |  |  |
| 502 | 165（12.9%） |  | 45(14.0%) |  | 210(13.1%) |  |  |
| 503 | 77（6.0%） |  | 18(5.6%) |  | 95(5.9%) |  |  |
| 504 | 432（33.6%） |  | 106(33.0%) |  | 538(33.5%) |  |  |
| 505 | 108（8.4%） |  | 29(9.1%) |  | 137(8.6%) |  |  |
| AJCC stage |  |  |  |  |  |  | 0.293 |
| Ⅰ | 279（21.7%） |  | 80（24.9%） |  | 359(22.4%) |  |  |
| Ⅱ | 740（57.6%） |  | 182（56.7%） |  | 922(57.4%) |  |  |
| Ⅲ | 197（15.3%） |  | 49（15.3%） |  | 246(15.3%) |  |  |
| Ⅳ | 68（5.4%） |  | 10（3.1%） |  | 78(4.9%) |  |  |
| T stage |  |  |  |  |  |  | 0.512 |
| T1 | 309（24.1%） |  | 90（28.0%） |  | 399(24.9%) |  |  |
| T2 | 624（48.6%） |  | 146(45.5%) |  | 770(48.0%) |  |  |
| T3 | 217（16.9%） |  | 54(16.8%) |  | 271(16.9%) |  |  |
| T4 | 134（10.4%） |  | 31(9.7%) |  | 165(10.3%) |  |  |
| N stage |  |  |  |  |  |  | 0.737 |
| N0 | 978（76.2%） |  | 245(76.3%) |  | 1223(76.2%) |  |  |
| N1 | 225(17.5%) |  | 51(15.9%) |  | 276(17.2%) |  |  |
| N2 | 54(4.2%) |  | 17(5.3%) |  | 71(4.4%) |  |  |
| N3 | 27(2.1%) |  | 8(2.5%) |  | 35(2.2%) |  |  |
| **Supplementary Table 1.** The characteristics of 1605 patients of MBC (Continued) | | | | | | | |
| M stage |  |  |  |  |  |  | 0.104 |
| M0 | 1216(94.7%) |  | 311(96.9%) |  | 1527(95.1%) |  |  |
| M1 | 68(5.3%) |  | 10(3.1%) |  | 78(4.9%) |  |  |
| ER status |  |  |  |  |  |  | 0.589 |
| Negative | 1002(78.0%) |  | 246(76.6%) |  | 1248(77.8%) |  |  |
| Positive | 282(22.0%) |  | 75(71.4%) |  | 357(22.2%) |  |  |
| PR status |  |  |  |  |  |  | 0.512 |
| Negative | 1106(86.1%) |  | 281(87.5%) |  | 1387(86.4%) |  |  |
| Positive | 178(13.9%) |  | 40(12.5%) |  | 218(13.6%) |  |  |
| HER-2 status |  |  |  |  |  |  | 0.397 |
| Negative | 1201(93.5%) |  | 296(92.2%) |  | 1497(93.3%) |  |  |
| Positive | 83(6.5%) |  | 25(7.8%) |  | 108(6.7%) |  |  |
| Subtype |  |  |  |  |  |  | 0.472 |
| HR+/HER2- | 324(25.2%) |  | 77(24.0%) |  | 401(25.0%) |  |  |
| HR+/HER2+ | 31(2.4%) |  | 6(1.9%) |  | 37(2.3%) |  |  |
| HR-/HER2+ | 52(4.0%) |  | 19(5.9%) |  | 71(4.4%) |  |  |
| HR-/HER2- | 877(68.3%) |  | 219(68.2%) |  | 1096(68.3%) |  |  |
| Surgery |  |  |  |  |  |  | 0.974 |
| no surgery | 63(4.9%) |  | 15(4.7%) |  | 78(4.9%) |  |  |
| breast-conserving | 537(41.8%) |  | 136(42.4%) |  | 67.3(41.9%) |  |  |
| mastectomy | 684(53.3%) |  | 170(53.0%) |  | 854(53.2%) |  |  |
| Chemotherapy |  |  |  |  |  |  | 0.337 |
| No | 424(33.0%) |  | 97(30.2%) |  | 521(32.5%) |  |  |
| Yes | 860(67.0%) |  | 224(69.8%) |  | 1084(67.5%) |  |  |
| Radiaotherapy |  |  |  |  |  |  | 0.122 |
| No | 634(49.4%) |  | 143(44.5%) |  | 777(48.4%) |  |  |
| Yes | 650(50.6%) |  | 178(55.5%) |  | 828(51.6%) |  |  |

Abbreviations: MBC Metaplastic breast carcinoma, IDC Infiltrating ductal carcinoma, 502 Upper-inner quadrant of breast, 503 Lower-inner quadrant of breast, 504 Upper-outer quadrant of breast, 505 Lower-outer quadrant of breast, ER Estrogen receptor, PR Progesterone receptor, HER-2 Human epidermal growth factor receptor 2
