# Supplementary material for: Clinical characteristics and overall survival prognostic nomogram for metaplastic breast cancer
Source: Front Oncol. 2023 Mar 2;13:1030124. doi: 10.3389/fonc.2023.1030124 (PMC10018193; doi:10.3389/fonc.2023.1030124)
Supplement: Supplementary file 2 [file Table_2.doc]

| **Supplementary Table2.** The characteristics of 1284 American patients and 49 Chinese patients of MBC. | | | | | | | |
| --- | --- | --- | --- | --- | --- | --- | --- |
| **Characteristic** | **Training cohort,**  **N(%)** (n=1284) |  | **Validation cohort Ⅱ, N(%)** (n=49) |  | ***χ***2 |  | ***P***-value |
| Age(years) |  |  |  |  | 30.78 |  | <0.001 |
| <50 | 267（20.8%） |  | 25 (51.0%) |  |  |  |  |
| 50-64 | 474（36.9%） |  | 18(36.7%) |  |  |  |  |
| 65-79 | 385（30.0%） |  | 6(12.3%) |  |  |  |  |
| 80+ | 158（12.3%） |  | 0(0%) |  |  |  |  |
| Sex |  |  |  |  | — |  | 1.000 |
| Female | 1278（99.5%） |  | 49(100.0%) |  |  |  |  |
| Male | 6（0.5%） |  | 0(0%) |  |  |  |  |
| Race |  |  |  |  | 401.19 |  | <0.001 |
| White | 968（75.4%） |  | 0(0%) |  |  |  |  |
| Black | 215（16.7%） |  | 0(0%) |  |  |  |  |
| Others | 101（7.9%） |  | 49(100.0%) |  |  |  |  |
| Marital |  |  |  |  | 39.49 |  | <0.001 |
| Married | 673（52.4%） |  | 48(98.0%) |  |  |  |  |
| Single | 248（19.3%） |  | 1(2.0%) |  |  |  |  |
| Divorced | 363（28.3%） |  | 0(0%) |  |  |  |  |
| Laterality |  |  |  |  | 0.01 |  | 0.975 |
| Right | 626（48.8%） |  | 24(49.0%) |  |  |  |  |
| Left | 658（51.2%） |  | 25(51.0%) |  |  |  |  |
| Site |  |  |  |  | 2.5 |  | 0.645 |
| others | 502（39.1%） |  | 17(34.7%) |  |  |  |  |
| 502 | 165（12.9%） |  | 6(12.2%) |  |  |  |  |
| 503 | 77（6.0%） |  | 3(6.1%) |  |  |  |  |
| 504 | 432（33.6%） |  | 21(42.9%) |  |  |  |  |
| 505 | 108（8.4%） |  | 2(4.1%) |  |  |  |  |
| AJCC stage |  |  |  |  | — |  | 0.933 |
| Ⅰ | 279（21.7%） |  | 10（20.4%） |  |  |  |  |
| Ⅱ | 740（57.6%） |  | 29（59.2%） |  |  |  |  |
| Ⅲ | 197（15.3%） |  | 8（16.3%） |  |  |  |  |
| Ⅳ | 68（5.4%） |  | 2（4.1%） |  |  |  |  |
| T stage |  |  |  |  | — |  | 0.941 |
| T1 | 309（24.1%） |  | 11（22.4%） |  |  |  |  |
| T2 | 624（48.6%） |  | 25(51.0%) |  |  |  |  |
| T3 | 217（16.9%） |  | 8(16.3%) |  |  |  |  |
| T4 | 134（10.4%） |  | 5(10.3%) |  |  |  |  |
| **Supplementary Table2.**  The characteristics of 1284 American patients and 49 Chinese patients of MBC (Continued) | | | | | | | |
| N stage |  |  |  |  | — |  | 0.068 |
| N0 | 978（76.2%） |  | 32(65.3%) |  |  |  |  |
| N1 | 225(17.5%) |  | 11(22.4%) |  |  |  |  |
| N2 | 54(4.2%) |  | 5(10.2%) |  |  |  |  |
| N3 | 27(2.1%) |  | 1(2.1%) |  |  |  |  |
| M stage |  |  |  |  | 0.14 |  | 0.708 |
| M0 | 1216(94.7%) |  | 47(95.9%) |  |  |  |  |
| M1 | 68(5.3%) |  | 2(4.1%) |  |  |  |  |
| ER status |  |  |  |  | 0.07 |  | 0.796 |
| Negative | 1002(78.0%) |  | 39(79.6%) |  |  |  |  |
| Positive | 282(22.0%) |  | 10(20.4%) |  |  |  |  |
| PR status |  |  |  |  | 0.01 |  | 0.933 |
| Negative | 1106(86.1%) |  | 42(85.7%) |  |  |  |  |
| Positive | 178(13.9%) |  | 7(14.3%) |  |  |  |  |
| HER-2 status |  |  |  |  | 0.22 |  | 0.636 |
| Negative | 1201(93.5%) |  | 45(91.8%) |  |  |  |  |
| Positive | 83(6.5%) |  | 4(8.2%) |  |  |  |  |
| Subtype |  |  |  |  | 0.55 |  | 0.908 |
| HR+/HER2- | 324(25.2%) |  | 12(24.5%) |  |  |  |  |
| HR+/HER2+ | 31(2.4%) |  | 2(4.1%) |  |  |  |  |
| HR-/HER2+ | 52(4.0%) |  | 2(4.1%) |  |  |  |  |
| HR-/HER2- | 877(68.3%) |  | 33(67.3%) |  |  |  |  |
| Surgery |  |  |  |  | 34.69 |  | <0.001 |
| no surgery | 63(4.9%) |  | 0(0%) |  |  |  |  |
| breast-conserving | 537(41.8%) |  | 2(4.1%) |  |  |  |  |
| mastectomy | 684(53.3%) |  | 47(95.9%) |  |  |  |  |
| Chemotherapy |  |  |  |  | 18.18 |  | <0.001 |
| No | 424(33.0%) |  | 2(4.1%) |  |  |  |  |
| Yes | 860(67.0%) |  | 47(95.9%) |  |  |  |  |
| Radiaotherapy |  |  |  |  | 2.65 |  | 0.104 |
| No | 634(49.4%) |  | 30(61.2%) |  |  |  |  |
| Yes | 650(50.6%) |  | 19(38.8%) |  |  |  |  |

Abbreviations: MBC Metaplastic breast carcinoma, IDC Infiltrating ductal carcinoma, 502 Upper-inner quadrant of breast, 503 Lower-inner quadrant of breast, 504 Upper-outer quadrant of breast, 505 Lower-outer quadrant of breast, ER Estrogen receptor, PR Progesterone receptor, HER-2 Human epidermal growth factor receptor 2
